# Synthesis and Tumor Cytotoxicity of Novel Amide Derivatives of β-Hederin

**DOI:** 10.3390/molecules15117871

**Published:** 2010-11-03

**Authors:** Yang Liu, Wen-Xiang Lu, Mao-Cai Yan, Yang Yu, Takashi Ikejima, Mao-Sheng Cheng

**Affiliations:** 1 Key Laboratory of Structure-Based Drug Design and Discovery (Ministry of Education), Shenyang Pharmaceutical University, Shenyang 110016, China; E-Mails: ly_99@sina.com (Y.L.); wenxiang0921@163.com (W.-X.L.); yanmaocai@126.com (M.-C.Y.); 2 China-Japan Research Institute of Medical Pharmaceutical Sciences, Shenyang Pharmaceutical University, Shenyang 110016, China; E-Mails: yy.yuyang@yahoo.com.cn (Y.Y.); ikejimat@vip.sina.com (T.I.)

**Keywords:** triterpenoid saponins, β-hederin, tumor cytotoxicity, synthesis

## Abstract

Thirteen novel triterpenoid saponins, designed as amide derivatives of the natural cytotoxic saponin β-hederin, were synthesized by a stepwise glycosylation strategy. The *in vitro* cytotoxic activity of these compounds was evaluated against five different tumor cell lines. Most of the evaluated compounds showed effective inhibitory activity against at least one tumor cell line at micromolar concentrations. The preliminary structure-activity relationships (SAR) indicate that mide derivatization at C-28 resulted in highly cytotoxic derivatives on specific tumor cell lines, and also resulted in an increase in the antitumor selectivity of β-hederin.

## 1. Introduction

**Figure 1:**
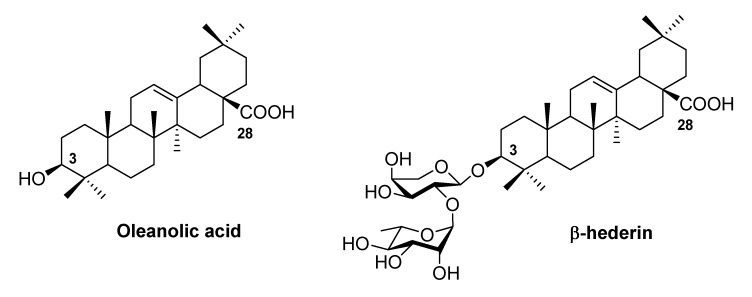
Structures of OA and β-hederin.

Oleanolic acid (OA, see Figure 1) is a pentacyclic triterpenoid widely distributed in Nature and possessing various important bioactivities, such as antitumor, anti-HIV, hepatoprotection, and anti-inflammatory properties[1,2,3]. OA also serves as an aglycon of many natural saponins, which display significantly higher levels of activity than OA itself. For instance, β-hederin (oleanolic acid 3-*O*-α-L-rhamnopyranosyl-(1→2)-α-L-arabinopyranoside, see Figure 1) [4], an OA glycoside that bears a unique disaccharide at C3-OH, shows excellent inhibitory activity against many tumor cells [5,6]. In our previous studies, the chemical synthesis of β-hederin and its glycosylated derivatives was completed, and their antitumor activity was evaluated [7,8]. 

Recently, some researchers have explored the structure-activity relationships of other pentacyclic triterpenic compounds, such as ursolic acid [9,10] and betulonic acid [11]. Those studies led to similar conclusions that the derivatives with a substitution of amino groups at C-28 often showed stronger cytotoxic antitumor activity. Therefore, we were inspired to investigate the possibility of similar properties in the medicinal chemistry of triterpenoid saponins. The present work describes the initial study of the synthesis of 13 novel amide derivatives of β-hederin and the evaluation of their cytotoxic activity against tumors. 

## 2. Results and Discussion

### 2.1. Synthesis

As far as β-hederin is concerned, the hydroxyl group at C3 is connected with a disaccharide moiety to form the so-called OA 3-glycosides. Therefore, only if the sugar substructure was well protected, can the free carboxyl group at C-28 be modified by various acylations. Based on our previous work [7,8,12], we developed a facile method to prepare the amide derivatives of β-hederin using OA, L-arabinose, L-rhamnose and some commercially available amines as the starting materials. 

**Scheme 1:**
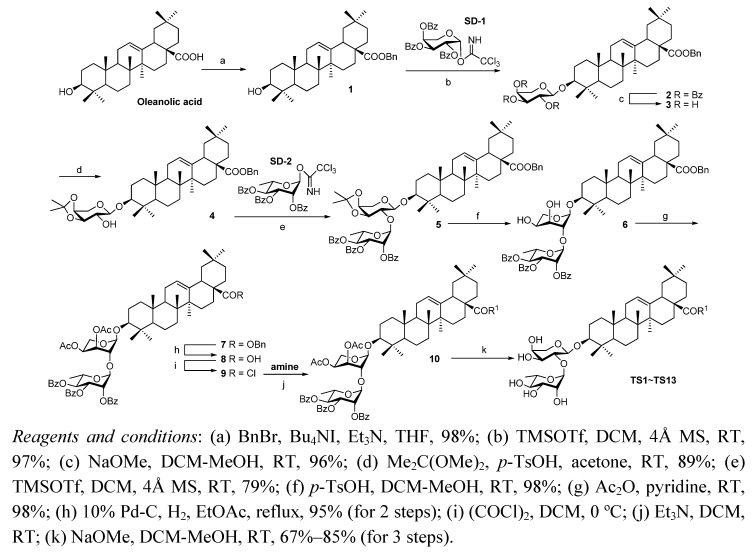
Synthesis of amide derivatives of β-hederin.

The compounds were synthesized as depicted in Scheme 1. To protect the carboxyl group of OA, benzyl ester **1** was prepared by the combination of OA, BnBr and K_2_CO_3_ in THF-H_2_O with Bu_4_NI as a phase transfer catalyst. Compound **1** was glycosylated with perbenzoylated arabinosyl trichloroacetimidate **SD-1** [13] under the promotion of TMSOTf to produce an excellent yield of compound **2**. Debenzoylation of compound **2** in the MeOH solution of NaOMe yielded saponin **3** without affecting the benzyl ester at C-28. Selective protection of 3-OH and 4-OH of the arabinose residue was successfully accomplished using 2,2-dimethoxypropane to furnish compound **4**, which was combined with perbenzoylated rhamnosyl trichloroacetimidate **SD-2** [14] under the same glycosylation conditions to generate compound **5** with a 79% yield. When the isopropylidene moiety was removed to prepare the intermediate **6**, the ^1^*C*_4_ conformation of the arabinose residue resulted produced via conformational inversion. Based on the ^1^H-NMR spectrum of compound **6**, the *J*_1'-2'_ value of the arabinose residue was changed to 1.3, much smaller than the normal value of the α-arabinosyl conformation (usually not less than 5.0). Moreover, the chemical shift (*δ*) of anomic C atom (109.3) is larger than that of the natural product (104.8). From the point of view of the chemical structure, the aglycone and perbenzoylated rhamnose connected with 1-OH and 2-OH of the arabinose residue, respectively, increased steric hindrance to such an extent that a chair inversion of arabinose resulted. Next, the two free hydroxyl groups were shielded by acetylation in pyridine solution to furnish compound **7**, whose benzyl group was easily removed through catalytic hydrogenation to produce compound **8** without affecting the double bond between C-12 and C-13 of OA. Compound **8 **was treated with oxalyl chloride to yield compound **9**, which then reacted with the appropriate amino compounds in the presence of Et_3_N to generate corresponding compounds **10**. Last, rapid and complete removal of all acyl groups in basic solution yielded the final products **TS1**-**TS13**. Fortunately, the arabinose ring returned to the normal ^4^*C*_1_ conformation after the final deprotection, which was confirmed by the ^1^H-NMR data. 

### 2.2. Biological evaluation

The preliminary *in vitro* biological evaluation was performed by a standard MTT assay to investigate the cytotoxicity of these saponins against five tumor cell lines: HeLa (cervical), MCF-7 (breast), HL-60 (leukemia), HT1080 (fibrosarcoma), and HepG2 (liver). The synthetic β-hederin was used as a reference compound. As shown in Table 1, although most of the evaluated compounds were found to be less active than β-hederin, they showed effective inhibitory activity against at least one tumor cell line at micromolar concentrations. It is worth emphasizing that compounds with a substitution of piperazine or methylpiperazine (**TS8** and **TS9**) displayed more potent activity than β-hederin. However, none of the evaluated compounds showed any toxicity towards the HT1080 or HepG2 cell lines within the investigated concentration range. The SAR indicated that the conversion of β-hederin into an amide yielded a moderately active derivative. The modified saponins with the aliphatic amine substructure seemed to display stronger activity than those bearing aromatic amines. Generally, the amide derivatization at C-28 resulted in highly cytotoxic derivatives on specific tumor cell lines, which means the antitumor selectivity of β-hederin was increased. 

**Table 1:** Structures and tumor cytotoxicity of amide derivatives of β-hederin.

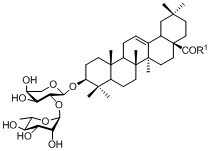						
Compd.	R^1^	IC_50_ (μM)				
		HeLa	MCF-7	HL-60	HT1080	HepG2
**TS1**	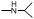	> 100	> 100	14.46	> 50	> 50
**TS2**	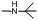	> 100	> 100	12.78	> 200	> 100
**TS3**	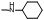	> 100	> 100	11.53	> 100	> 100
**TS4**	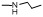	24.14	> 100	> 100	> 200	> 100
**TS5**	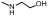	> 100	19.72	> 100	> 100	> 100
**TS6**	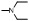	> 100	13.80	> 100	> 50	> 50
**TS7**	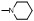	> 100	> 100	15.58	> 50	> 50
**TS8**	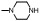	8.80	9.89	> 100	> 50	> 50
**TS9**	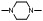	4.74	17.13	> 100	> 100	> 100
**TS10**	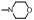	> 100	> 100	18.20	> 100	> 100
**TS11**	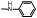	> 200	> 100	> 100	> 100	> 100
**TS12**	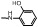	> 100	> 100	16.29	> 100	> 100
**TS13**	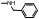	18.99	14.83	> 100	> 50	> 50
**β-hederin**	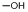	12.47	9.53	8.26	> 50	19.72

## 3. Experimental

### 3.1. General

Commercial reagents were used without further puriﬁcation unless otherwise stated. Solvents were dried and redistilled prior to use in the usual way. Analytical TLC was performed on silica gel HF_254_ plates. Preparative column chromatography (CC) was performed with silica gel H. Melting points were measured with a Büchi B-540 Melting Point apparatus. Optical rotations were measured at the sodium D-line at room temperature (RT) with a Perkin–Elmer 241MC polarimeter. ^1^H- and ^13^C-NMR spectra were recorded on a Bruker Avance AV600 MHz spectrometer using Me_4_Si as the internal standard. HRMS spectra were recorded on a high resolution ESI-FTICR mass spectrometer.

### 3.2. Benzyl oleanolate 3-O-3,4-O-isopropylidene-α-L-arabinopyranoside (***4***)

A suspension of OA (1.00 g, 2.2 mmol), BnBr (0.42 mL, 3.5 mmol), K_2_CO_3_ (0.60 g, 4.4 mmol) and Bu_4_NI (0.08 g, 0.22 mmol) in 40:1 THF-H_2_O (41 mL) was stirred overnight at RT. The mixture was then filtered, and the filtrate was concentrated under vacuum and purified by silica gel column chromatography (8:1 petroleum ether–EtOAc) to give oleanolic acid benzyl ester **1** (1.17 g, 98%) as a white amorphous solid. Compound **1** (1.00 g, 1.83 mmol), trichloroacetimidate **SD-1** (1.27 g, 2.10 mmol) and powdered 4Å molecular sieves (MS, 500 mg) were mixed in dry DCM (25 mL) and stirred at RT for 20 min. A dry DCM solution (2.0 mL) of TMSOTf (0.02 mL, 0.01 mmol) was then added dropwise and the mixture was stirred for approximately 1.5 h until the reagents were completely consumed. The mixture was neutralized with Et_3_N (0.20 mL) and filtered. The filtrate was concentrated and purified by CC (8:1 petroleum ether–EtOAc) to generate compound **2 **(1.87 g, 97%) as a white foam. A fresh solution of NaOMe in MeOH (1.0 mol/L, 1.70 mL) was added to a solution of **2** (1.50 g, 1.50 mmol) in 1:2 DCM–MeOH (40 mL). The mixture was stirred at RT for 2 h, neutralized with Dowex H^+^ resin, filtered, the filtrate was concentrated, and the residue subjected to CC (EtOAc) to yield saponin **3** (991 mg, 96%) as a white amorphous solid. Me_2_C(OMe)_2_ (0.31 mL, 2.50 mmol) and *p*-TsOH (17.2 mg) was added to a solution of **3** (679 mg, 1.00 mmol) in dry acetone (10 mL). The mixture was stirred for 4 h before Et_3_N (0.20 mL) was added. The solution was concentrated and purified by CC (6:1 petroleum ether–EtOAc) to generate afford compound **4** (634 mg, 89%) as a white foam. [α]^25^_D_ +45.0 (*c* 1.60, CHCl_3_); ^1^H-NMR (CDCl_3_): *δ *7.34 (m, 5H, Ar-H), 5.28 (t, *J* = 3.0 Hz, 1H, H-12), 5.07 (dd, *J* = 18.7, 12.6 Hz, 2H, PhC*H*_2_), 4.22–4.17 (m, 3H, H-1', H-4', H-5'-1), 4.06 (dd, *J* = 7.8, 6.1 Hz, 1H, H-3'), 3.75 (dd, *J* = 13.9, 3.5 Hz, 1H, H-5'-2), 3.63 (dd, *J* = 7.8, 7.8 Hz, 1H, H-2'), 3.12 (dd, *J* = 11.5, 4.6 Hz, 1H, H-3), 2.91 (dd, *J* = 13.8, 3.3 Hz, 1H, H-18), 2.30 (br s, 1H, O*H*), 1.54, 1.36 (s each, 3H each, O-(C*H*_3_)_2_C-O), 1.11, 0.98, 0.92, 0.89, 0.88, 0.82, 0.60 (s each, 3H each, 7×Me); HRMS: calcd for C_38_H_59_O_7_ (M-Bn): 627.4261; found: m/z 627.4255.

### 3.3. Benzyl oleanolate 3-O-2,3,4-tri-O-benzoyl-α-L-rhamnopyranosyl-(1→2)-3,4-O-isopropylidene-α-L-arabinopyranoside (***5***)

A mixture of compound **4** (560 mg, 0.78 mmol), trichloroacetimidate **SD-2** (630 mg, 1.00 mmol) and powdered 4Å MS (300 mg) in dry DCM (10 mL) was stirred at RT for 20 min. A dry DCM solution of TMSOTf (0.005 mmol) was added dropwise, and the mixture was stirred for 2 h, followed by the addition of Et_3_N (0.20 mL) and filtration. The ﬁltrate was concentrated and subjected to CC (8:1 petroleum ether–EtOAc) to furnish disaccharide saponin **5** (730 mg, 79%) as a white foam. [α]^25^_D_ +96.7 (*c* 2.58, CHCl_3_); ^1^H-NMR (CDCl_3_): *δ* 8.12–7.21 (m, 20H, Ar-H), 5.87 (dd, *J* = 10.2, 3.3 Hz, 1H, H-3"), 5.76 (s, 1H, H-1"), 5.65 (m, 2H, H-2", H-4"), 5.30 (t, *J* = 3.0 Hz, 1H, H-12), 5.07 (dd, *J* = 22.4, 12.6 Hz, 2H, PhC*H*_2_), 4.53 (m, 1H, H-5"), 4.47 (d, *J* = 3.0 Hz, 1H, H-1'), 4.25 (m, 2H, H-3', H-4'), 4.17 (m, 1H, H-5'-1), 3.90 (dd, *J* = 3.0, 3.0 Hz, 1H, H-2'), 3.79 (m, 1H, H-5'-2), 3.17 (dd, *J* = 11.3, 4.1 Hz, 1H, H-3), 2.92 (m, 1H, H-18), 1.55, 1.35 (s each, 3H each, O-(C*H*_3_)_2_C-O), 1.34 (d, *J* = 6.1 Hz, 3H, H-6"), 1.14, 0.95, 0.93, 0.92, 0.90, 0.89, 0.64 (s each, 3H each, 7×Me); HRMS: calcd for C_65_H_81_O_14_ (M-Bn): 1085.5626; found: m/z 1085.5619.

### 3.4. Benzyl oleanolate 3-O-2,3,4-tri-O-benzoyl-α-L-rhamnopyranosyl-(1→2)-α-L-arabinopyranoside (***6***)

Compound **5** (705 mg, 0.60 mmol) was dissolved in DCM–MeOH (1:2, 40 mL), and then *p*-TsOH (78 mg) was added. The solution was stirred at RT for 3 h, after which Et_3_N (0.40 mL) was added, and the mixture was concentrated and puriﬁed by CC (2:1 petroleum ether–EtOAc) to yield compound **6** (666 mg, 98%) as a white amorphous solid. [α]^25^_D_ +77.3 (*c* 2.27, CHCl_3_); ^1^H-NMR (CDCl_3_): *δ* 8.10–7.23 (m, 20H, Ar-H), 5.84 (dd, *J* = 10.2, 3.1 Hz, 1H, H-3"), 5.65 (m, 2H, H-2", H-4"), 5.36 (s, 1H, H-1"), 5.29 (br s, 1H, H-12), 5.07 (dd, *J* = 18.7, 12.6 Hz, 2H, PhC*H*_2_), 4.81 (d, *J* = 1.3 Hz, 1H, H-1'), 4.34 (m, 1H, H-5"), 4.11–3.98 (m, 3H, H-2', H-4', O*H*), 3.82 (m, 1H, H-5'-1), 3.67 (m, 1H, H-5'-2), 3.45 (d, *J* = 7.9 Hz, 1H, H-3'), 3.18 (dd, *J* = 11.0, 3.2 Hz, 1H, H-3), 2.91 (m, 1H, H-18), 2.52 (br s, 1H, O*H*), 1.34 (d, *J* = 6.0 Hz, 3H, H-6"), 1.12, 1.05, 0.92, 0.89, 0.88, 0.84, 0.61 (s each, 3H each, 7×Me); HRMS: calcd for C_62_H_77_O_14_ (M-Bn): 1045.5313; found: m/z 1045.5307.

### 3.5. Oleanolic acid 3-O-2,3,4-tri-O-benzoyl-α-L-rhamnopyranosyl-(1→2)-3,4-di-O-acetyl-α-L-arabinopyranoside (***8***)

A solution of compound **6** (600 mg, 0.53 mmol) and Ac_2_O (0.25 mL, 2.65 mmol) in dry pyridine (5 mL) was stirred at RT overnight. The solvent was evaporated in a vacuum, and the resulting residue was dissolved in DCM (20 mL), washed with water (15 mL × 3), and dried over MgSO_4_. The mixture was filtered, and the filtrate was concentrated to produce crude compound **7** as a white amorphous solid, which was then dissolved in dry EtOAc (20 mL). After adding in 100 mg of 10% Pd–C, the solution was refluxed and bubbled up with H_2 _(25 mL/min) for 5 h. Pd–C was removed through filtration, and the filtrate was concentrated to dryness, which was puriﬁed by CC (3:1 petroleum ether–EtOAc) to generate compound **8** (569 mg, 95%, for the 2 steps) as a white amorphous solid. [α]^25^_D_ +57.2 (*c* 1.68, CHCl_3_); ^1^H-NMR (CDCl_3_): *δ *8.17–7.27 (m, 15H, Ar-H), 5.85 (dd, *J* = 10.2, 3.3 Hz, 1H, H-3"), 5.57 (s, 1H, H-1"), 5.65 (m, 2H, H-2", H-4"), 5.28 (t, *J* = 3.0 Hz, 1H, H-12), 4.77 (d, *J* = 3.0 Hz, 1H, H-1'), 4.53 (m, 1H, H-5"), 4.25–4.21 (m, 2H, H-3', H-4'), 4.18 (m, 1H, H-5'-1), 3.93 (m, 1H, H-2'), 3.71 (m, 1H, H-5'-2), 3.16 (dd, *J* = 11.3, 4.1 Hz, 1H, H-3), 2.94 (m, 1H, H-18), 1.94 (s, 6H, 2×C*H*_3_CO), 1.37 (d, *J* = 6.0 Hz, 3H, H-6"), 1.19, 1.15, 1.06, 0.90, 0.89, 0.83, 0.78 (s each, 3H each, 7×Me); HRMS: calcd for C_62_H_77_O_14_ (M+Na): 1155.5603; found: m/z 1155.5597.

### 3.6. General procedure for the synthesis of oleanolic amide 3-O-α-L-rhamnopyranosyl-(1→2)-α-L-arabinopyranosides

Oxalyl chloride (0.37 mL, 4.40 mmol) was added dropwise to a solution of compound **8** (500 mg, 0.44 mmol) in redistilled DCM (10 mL). The mixture was stirred at RT for 6 h. Then, the solvents were co-evaporated with toluene for complete removal of the excess oxalyl chloride to yield compound **9** as a yellow foam. A solution of compound **9** in redistilled DCM (10 mL) was treated with the corresponding amine (0.80 mmol) and 5 drops of Et_3_N and stirred for 4 h under nitrogen. The mixture was washed with satd aq NaCl (10 mL × 3), dried over MgSO_4_, and concentrated *in vacuo* to yield the crude product **10 **which was dissolved in dry 1:2 DCM–MeOH (12 mL) and treated with a fresh solution of NaOMe in MeOH (1.0 mol/L, 1.00 mL). The solution was stirred at RT for 2 h, neutralized with Dowex H^+^ resin to pH 7, and filtered. The filtrate was concentrated and subjected to CC (20:10:1 CHCl_3_–MeOH–H_2_O) to give the target saponins in yields of 67%–85% (for the 3 steps). 

#### 3.6.1. N-isopropyloleanolic amide 3-O-α-L-rhamnopyranosyl-(1→2)-α-L-arabinopyranoside (***TS1***)

White powder, m.p. 176.4–178.3 ºC; [α]^25^_D_ +11.5 (*c* 0.13, CH_3_OH); ^1^H-NMR (pyridine-*d*_5_): *δ* 6.92 (d, 1H, *J* = 7.2 Hz, N*H*), 6.12 (s, 1H, H-1''), 5.43 (br s, 1H, H-12), 4.90 (d, 1H, *J* = 5.4 Hz, H-1'), 4.72 (m, 1H, H-2''), 4.62–4.54 (m, 3H, H-2', H-3'', H-5''), 4.34–4.27 (m, 5H, H-3', H-4', H-5'-1, H-4'', NC*H*(CH_3_)_2_), 3.82 (m, 1H, H-5'-2), 3.25 (dd, 1H, *J* = 11.3, 4.1 Hz, H-3), 3.01 (br d, 1H, *J* = 13.8 Hz, H-18), 1.63 (d, 3H, *J* = 6.5 Hz, H-6''), 1.26, 1.20, 1.75, 1.16, 1.08, 0.96, 0.92, 0.91, 0.86 (s each, 3H each, 9×Me); ^13^C-NMR(pyridine-*d*_5_): *δ* 176.5 (C-28), 144.9 (C-13), 122.6 (C-12), 104.9 (C-1'), 101.7 (C-1''), 88.7 (C-3), 75.8 (C-2'), 74.1, 74.1 (C-3', C-4''), 72.6, 72.4 (C-2'', C-3''), 69.9 (C-5''), 68.9 (C-4'), 64.9 (C-5'), 55.8 (C-5), 47.9 (C-9), 46.7 (C-17), 46.1 (C-19), 42.2 (C-14), 41.8 (C-18), 41.4 (C-NH), 39.8 (C-4), 39.5 (C-8), 38.9 (C-1), 37.0 (C-10), 34.4 (C-21), 33.8 (C-29), 33.2 (C-7), 33.1 (C-22), 30.8 (C-20), 28.0, 27.8 (C-15, C-23), 26.5 (C-2), 26.0 (C-27), 23.7, 23.7, 23.5 (C-30, C-11, C-16), 22.9, 22.3 (CH(*C*H_3_)_2_), 18.6, 18.6 (C-6, C-6''), 17.3 (C-26), 17.0 (C-24), 15.7 (C-25); HRMS: calcd for C_44_H_73_NO_1__0_ (M+Na): 798.5232; found: m/z 798.5227. 

#### 3.6.2. N-tert-butyloleanolic amide 3-O-α-L-rhamnopyranosyl-(1→2)-α-L-arabinopyranoside (***TS2***)

White powder, m.p. 187.5–189.3 ºC; [α]^25^_D_ +7.5 (*c* 0.24, CH_3_OH); ^1^H-NMR (pyridine-*d*_5_): *δ* 7.15 (d, 1H, *J* = 7.0 Hz, N*H*), 6.12 (s, 1H, H-1''), 5.43 (br s, 1H, H-12), 4.89 (d, 1H, *J* = 5.3 Hz, H-1'), 4.73 (m, 1H, H-2''), 4.64–4.54 (m, 3H, H-2', H-3'', H-5''), 4.33–4.27 (m, 4H, H-3', H-4', H-5'-1, H-4''), 3.81 (m, 1H, H-5'-2), 3.24 (dd, 1H, *J* = 11.5 Hz, 4.0, H-3), 2.87 (br d, 1H, *J* = 13.8 Hz, H-18), 1.62 (d, 3H, *J* = 6.1 Hz, H-6''), 1.44 (s, 9H, *t*-Bu), 1.25, 1.17, 1.10, 0.98, 0.91, 0.89, 0.88 (s each, 3H each, 7×Me); ^13^C- NMR (pyridine-*d*_5_): *δ* 176.8 (C-28), 145.0 (C-13), 122.7 (C-12), 104.9 (C-1'), 101.7 (C-1''), 88.7 (C-3), 75.9 (C-2'), 74.1, 73.9 (C-3', C-4''), 72.6, 72.4 (C-2'', C-3''), 69.9 (C-5''), 68.7 (C-4'), 64.8 (C-5'), 55.9 (C-5), 50.7 (C-NH), 47.9 (C-9), 47.0 (C-17), 46.8 (C-19), 42.4 (C-14), 42.3 (C-18), 39.8 (C-4), 39.5 (C-8), 39.0 (C-1), 37.0 (C-10), 34.5 (C-21), 33.5 (C-29), 33.2 (C-7), 33.0 (C-22), 30.8 (C-20), 28.8 (C(*C*H_3_)_3_), 28.1, 27.8 (C-15, C-23), 26.6 (C-2), 25.8 (C-27), 23.9, 23.9, 23.7 (C-30, C-11, C-16), 18.6, 18.6 (C-6'', C-6), 18.0 (C-26), 17.0 (C-24), 15.7 (C-25); HRMS: calcd for C_4__5_H_75_NO_10_ (M+Na): 812.5289; found: m/z 812.5285.

#### 3.6.3. N-cyclohexyloleanolic amide 3-O-α-L-rhamnopyranosyl-(1→2)-α-L-arabinopyranoside (***TS3***)

White powder, m.p. 201.5–203.7 ºC; [α]^25^_D_ +27.1 (*c* 0.30, CH_3_OH); ^1^H-NMR (pyridine-*d*_5_): *δ* 6.71 (d, 1H, *J* = 7.5 Hz, N*H*), 6.14 (s, 1H, H-1''), 5.46 (br s, 1H, H-12), 4.90 (d, 1H, *J* = 5.3 Hz, H-1'), 4.74 (m, 1H, H-2''), 4.65–4.55 (m, 3H, H-2', H-3'', H-5''), 4.33–4.28 (m, 4H, H-3', H-4', H-5'-1, H-4''), 4.06 (m, 1H, NC*H*), 3.82 (m, 1H, H-5'-2), 3.25 (dd, 1H, *J* = 11.3, 3.8 Hz, H-3), 3.09 (br d, 1H, *J* = 9.8 Hz, H-18), 2.12–1.78 (m, 10H, hexanyl), 1.62 (d, 3H, *J* = 6.1 Hz, H-6''), 1.27, 1.18, 1.09, 0.98, 0.95, 0.93, 0.89 (s each, 3H each, 7×Me); ^13^C-NMR (pyridine-*d*_5_): *δ* 176.5 (C-28), 145.0 (C-13), 122.7 (C-12), 104.9 (C-1'), 101.7 (C-1''), 88.8 (C-3), 75.9 (C-2'), 74.1, 73.9 (C-3', C-4''), 72.5, 72.4 (C-2'', C-3''), 69.9 (C-5''), 68.8 (C-4'), 64.8 (C-5'), 55.9 (C-5), 48.6 (C-NH), 48.0 (C-9), 46.8 (C-17), 46.3 (C-19), 42.3 (C-14), 41.9 (C-18), 39.8 (C-4), 39.5 (C-8), 38.9 (C-1), 37.0 (C-10), 34.5 (C-21), 33.6 (C-29), 33.2 (C-7), 33.0 (C-22), 30.9 (C-20), 28.1, 27.9 (C-15, C-23), 26.5 (C-2), 26.0 (C-27), 25.9, 25.6, 25.5 (hexanyl), 23.8, 23.8, 23.7 (C-30, C-11, C-16), 18.6, 18.6 (C-6'', C-6), 17.8 (C-26), 17.0 (C-24), 15.6 (C-25); HRMS: calcd for C_4__7_H_77_NO_10_ (M+Na): 838.5547; found: m/z 838.5544.

#### 3.6.4. N-propyloleanolic amide 3-O-α-L-rhamnopyranosyl-(1→2)-α-L-arabinopyranoside (***TS4***)

White powder, m.p. 178.6–181.0 ºC; [α]^25^_D_ +17.7 (*c* 0.27, CH_3_OH); ^1^H-NMR (pyridine-*d*_5_): *δ* 7.31 (m, 1H, N*H*), 6.15 (s, 1H, H-1''), 5.39 (br s, 1H, H-12), 4.88 (d, 1H, *J = *5.4 Hz, H-1'), 4.73 (m, 1H, H-2''), 4.63–4.55 (m, 3H, H-2', H-3'', H-5''), 4.34–4.27 (m, 4H, H-3', H-4', H-5'-1, H-4''), 3.80 (m, 1H, H-5'-2), 3.48–3.42 (m, 1H, NC*H*_2_CH_2_CH_3_), 3.27–3.21 (m, 2H, H-3, NC*H*_2_CH_2_CH_3_), 3.07 (br d, 1H, *J* = 13.0 Hz, H-18), 1.61 (d, 3H, *J = *5.4 Hz, H-6''), 1.58–1.54 (m, 2H, NCH_2_C*H*_2_CH_3_), 1.24, 1.15, 1.07, 0.91, 0.90, 0.89, 0.86 (s each, 3H each, 7×Me), 0.84–0.81 (t, 3H, NCH_2_CH_2_C*H*_3_); ^13^C-NMR (pyridine-*d*_5_): *δ* 177.4 (C-28), 144.9 (C-13), 122.7 (C-12), 104.9 (C-1'), 101.7 (C-1''), 88.7 (C-3), 75.8 (C-2'), 74.1, 74.0 (C-3', C-4''), 72.6, 72.4 (C-2'', C-3''), 69.9 (C-5''), 68.8 (C-4'), 64.8 (C-5'), 55.8 (C-5), 47.9 (C-9), 46.8 (C-17), 46.4 (C-19), 42.2, 41.9, 41.6 (N*C*H_2_CH_2_CH_3_, C-14, C-18), 39.7 (C-4), 39.5 (C-8), 38.8 (C-1), 37.0 (C-10), 34.4 (C-21), 33.8 (C-29), 33.2 (C-7), 33.0 (C-22), 30.9 (C-20), 28.0, 27.8 (C-15, C-23), 26.5 (C-2), 26.1 (C-27), 23.8 (C-30), 23.7 (C-11), 23.7 (C-16), 23.4 (NCH_2_*C*H_2_CH_3_), 18.6, 18.5 (C-6, C-6''), 17.5 (C-26), 17.0 (C-24), 15.6 (C-25), 11.8 (NCH_2_CH_2_*C*H_3_); HRMS: calcd for C_4__4_H_73_NO_10_ (M+Na): 798.5234; found: m/z 798.5230.

#### 3.6.5. N-(2-hydroxyethyl)oleanolic amide 3-O-α-L-rhamnopyranosyl-(1→2)-α-L-arabinopyranoside (***TS5***)

White powder, m.p. 184.6–187.3ºC; [α]^25^_D_ +0.3 (*c *0.30, CH_3_OH); ^1^H-NMR (pyridine-*d*_5_): *δ* 7.51 (m, 1H, N*H*), 6.13 (s, 1H, H-1''), 5.42 (br s, 1H, H-12), 4.90 (d, 1H, *J = *5.4 Hz, H-1'), 4.74 (m, 1H, H-2''), 4.62–4.54 (m, 3H, H-2', H-3'', H-5''), 4.32–4.28 (m, 4H, H-3', H-4', H-5'-1, H-4''), 4.02–4.00 (m, 2H, NCH_2_C*H*_2_OH), 3.89–3.87 (m, 1H, NC*H*_2_CH_2_OH), 3.86–3.82 (m, 1H, H-5'-2), 3.65–3.59 (m, 1H, NC*H*_2_CH_2_OH), 3.26 (dd, 1H, *J* = 11.1, 4.1 Hz, H-3), 3.06 (br d, 1H, *J* = 12.8 Hz, H-18), 1.62 (d, 3H, *J = *6.6 Hz, H-6''), 1.26, 1.22, 1.07, 0.96, 0.91, 0.90, 0.86 (s each, 3H each, 7×Me); ^13^C-NMR (pyridine-*d*_5_): *δ* 178.1 (C-28), 144.7 (C-13), 122.9 (C-12), 104.9 (C-1'), 101.7 (C-1''), 88.7 (C-3), 75.8 (C-2'), 74.0, 74.0 (C-3', C-4''), 72.6, 72.4 (C-2'', C-3''), 69.8 (C-5''), 68.8 (C-4'), 64.9 (C-5'), 61.4 (NCH_2_*C*H_2_OH), 55.8(C-5), 47.9 (C-9), 46.7 (C-17), 46.4 (C-19), 43.0 (N*C*H_2_CH_2_OH), 42.1, 41.9 (C-14, C-18), 39.7 (C-4), 39.5 (C-8), 38.8 (C-1), 36.9 (C-10), 34.3 (C-21), 33.6 (C-29), 33.1 (C-7), 32.8 (C-22), 30.9 (C-20), 28.0, 27.8 (C-15, C-23), 26.5, 26.1 (C-2, C-27), 23.8, 23.7, 23.7 (C-30, C-11, C-16), 18.6, 18.5 (C-6, C-6''), 17.3, 17.0 (C-26, C-24), 15.5(C-25); HRMS: calcd for C_4__3_H_71_NO_1__1_ (M+Na): 800.5027; found: m/z 800.5022.

#### 3.6.6. N,N-diethyloleanolic amide 3-O-α-L-rhamnopyranosyl-(1→2)-α-L-arabinopyranoside (***TS6***)

White powder, m.p. 159.4–161.2 ºC; [α]^25^_D_ -20.0 (*c* 0.14, CH_3_OH); ^1^H-NMR (pyridine-*d*_5_): *δ* 6.17 (s, 1H, H-1''), 5.43 (br s, 1H, H-12), 4.90 (d, 1H, *J* = 5.4 Hz, H-1'), 4.75 (m, 1H, H-2''), 4.64–4.56 (m, 3H, H-2', H-3'', H-5''), 4.32–4.27 (m, 4H, H-3', H-4', H-5'-1, H-4''), 3.83 (m, 1H, H-5'-2), 3.41–3.26 (m, 6H, H-3, H-18, N(C*H*_2_CH_3_)_2_), 1.63 (d, 3H, *J* = 6.6 Hz, H-6''), 1.27, 1.18, 1.14, 1.14, 1.09, 0.96, 0.93, 0.93, 0.88 (s each, 3H each, 9×Me); ^13^C-NMR (pyridine-*d*_5_): *δ* 175.0 (C-28), 145.6 (C-13), 121.8 (C-12), 105.0 (C-1'), 101.8 (C-1''), 88.9 (C-3), 76.0 (C-2'), 74.2, 74.0 (C-3', C-4''), 72.7, 72.5 (C-2'', C-3''), 70.0 (C-5''), 68.9 (C-4'), 64.9 (C-5'), 56.1 (C-5), 48.2 (C-9), 47.8 (C-17), 47.2 (C-19), 44.2 (N*C*H_2_CH_3_), 42.3, 42.3 (C-14, C-18), 39.8 (C-4), 39.6 (C-8), 38.9 (C-1), 37.2 (C-10), 34.5 (C-21), 33.6 (C-29), 33.3, 33.3 (C-7, C-22), 30.6 (C-20), 30.4 (N*C*H_2_CH_3_), 28.6, 28.2 (C-15, C-23), 26.7 (C-2), 26.0 (C-27), 24.3, 23.9, 22.9 (C-30, C-11, C-16), 18.6 (C-6, C-6''), 17.7 (C-26), 17.2 (C-24), 15.8 (C-25), 13.7, 13.7 (N(CH_2_*C*H_3_)_2_); HRMS: calcd for C_4__5_H_75_NO_10_ (M+Na): 812.5289; found: m/z 812.5281.

#### 3.6.7. 1-(piperidin-1-yl)ole-28-one 3-O-α-L-rhamnopyranosyl-(1→2)-α-L-arabinopyranoside (***TS7***)

White powder, m.p. 174.0–176.3ºC; [α]^25^_D_ -12.5 (*c *0.12, CH_3_OH); ^1^H-NMR (pyridine-*d*_5_): *δ* 6.17 (s, 1H, H-1''), 5.42 (br s, 1H, H-12), 4.89 (d, 1H, *J = *5.4 Hz, H-1'), 4.75 (m, 1H, H-2''), 4.64–4.56 (m, 3H, H-2', H-3'', H-5''), 4.32–4.27 (m, 4H, H-3', H-4', H-5'-1, H-4''), 3.81 (d, 1H, *J = *11.4 Hz, H-5'-2), 3.60–3.54 (m, 4H, H-piperidin-2,6), 3.40 (dd, 1H, *J* = 11.9, 4.2 Hz, H-3), 3.25 (br d, 1H, *J* = 13.0 Hz, H-18), 1.50 (m, 6H, H-piperidin-3,4,5), 1.62 (d, 3H, *J = *6.6 Hz, H-6''), 1.27, 1.18, 1.09, 0.95, 0.94, 0.94, 0.88 (s each, 3H each, 7×Me); ^13^C-NMR (pyridine-*d*_5_): *δ* 174.5 (C-28), 145.5 (C-13), 121.8 (C-12), 105.0 (C-1'), 101.8 (C-1''), 88.8 (C-3), 75.9 (C-2'), 74.1, 73.9 (C-3', C-4''), 72.6, 72.5 (C-2'', C-3''), 69.9 (C-5''), 68.8 (C-4'), 64.8 (C-5'), 56.1 (C-5), 48.1, 47.6, 46.9, 46.7 (C-9, C-piperidine, C-17, C-19), 44.2 (C-piperidine), 42.3, 42.3 (C-14, C-18), 39.6 (C-4), 39.5 (C-8), 38.9 (C-1), 37.1 (C-10), 34.3 (C-21), 33.4 (C-29), 33.2, 33.2 (C-7, C-22), 30.6 (C-20), 30.1 (C-piperidine), 28.4, 28.1 (C-15, C-23), 26.5, 26.5 (C-2, C-27), 26.2, 25.1 (C-piperidine), 24.2, 23.8, 22.9 (C-30, C-11, C-16), 18.6 (C-6, C-6''), 17.3, 17.1 (C-26, C-24), 15.7 (C-25); HRMS: calcd for C_4__6_H_75_NO_10_ (M+Na): 824.5389; found: m/z 824.5386.

#### 3.6.8. 1-(piperazin-1-yl)ole-28-one 3-O-α-L-rhamnopyranosyl-(1→2)-α-L-arabinopyranoside (***TS8***)

White powder, m.p. 230.3–231.1ºC; [α]^25^_D_ -19.4 (*c *0.32, CH_3_OH); ^1^H-NMR (pyridine-*d*_5_): *δ* 6.17 (s, 1H, H-1''), 5.41 (br s, 1H, H-12), 4.90 (d, 1H, *J = *5.4 Hz, H-1), 4.75 (m, 1H, H-2''), 4.65–4.56 (m, 3H, H-2', H-3'', H-5''), 4.33–4.28 (m, 4H, H-3', H-4', H-5'-1, H-4''), 4.28–4.20 (m, 4H, H-piperizine-2,6), 3.82 (d, 1H, *J = *11.4 Hz, H-5'-2), 3.43–3.77 (m, 5H, H-3, H-piperizine-3,5), 3.25 (br d, 1H, *J* = 13.3 Hz, H-18), 1.63 (d, 3H, *J = *6.0 Hz, H-6''), 1.24, 1.19, 1.11, 0.94, 0.93, 0.88, 0.85 (s each, 3H each, 7×Me); ^13^C-NMR (pyridine-*d*_5_): *δ* 175.3 (C-28), 145.0 (C-13), 122.0 (C-12), 104.9 (C-1'), 101.7 (C-1''), 88.7 (C-3), 75.9 (C-2'), 74.1, 74.0 (C-3', C-4''), 72.6, 72.4 (C-2'', C-3''), 69.9 (C-5''), 68.8 (C-4'), 64.9 (C-5'), 56.0 (C-5), 48.1 (C-9), 47.6, 46.7 (C-17, C-19), 44.4, 44.0, 44.0 (C-piperazine), 42.2, 42.2 (C-14, C-18), 39.5, 39.5, 38.9 (C-4, C-8, C-1), 37.1 (C-10), 34.1 (C-21), 33.1, 33.1, 33.1 (C-7, C-29, C-22), 30.5 (C-20), 30.2 (C-piperazine), 28.3, 28.1 (C-15, C-23), 26.5 (C-2), 26.1 (C-27), 24.1, 23.7, 22.7 (C-30, C-11, C-16), 18.6 (C-6, C-6''), 17.1, 17.0 (C-26, C-24), 15.6 (C-25); HRMS: calcd for C_4__5_H_74_N_2_O_10_ (M+Na): 825.5343; found: m/z 825.5336.

#### 3.6.9. 1-(4-methylpiperazin-1-yl)ole-28-one 3-O-α-L-rhamnopyranosyl-(1→2)-α-L-arabinopyranoside (***TS9***)

White powder, m.p. 190.2–192.7ºC; [α]^25^_D_ -17.5 (*c *0.16, CH_3_OH); ^1^H-NMR (pyridine-*d*_5_): *δ* 6.20 (s, 1H, H-1''), 5.42 (br s, 1H, H-12), 4.89 (d, 1H, *J = *5.4 Hz, H-1'), 4.77 (m, 1H, H-2''), 4.66–4.58 (m, 3H, H-2', H-3'', H-5''), 4.34–4.28 (m, 4H, H-3', H-4', H-5'-1, H-4''), 3.83–3.74 (m, 5H, H-5'-2, H-piperizine-2,6), 3.40 (dd, 1H, *J* = 11.7, 4.0 Hz, H-3), 3.25 (br d, 1H, *J* = 13.1 Hz, H-18), 2.33 (m, 4H, H-piperizine-3,5), 2.17 (s, 3H, NC*H*_3_), 1.63 (d, 3H, *J = *6.6 Hz, H-6''), 1.26, 1.19, 1.10, 0.95, 0.94, 0.93, 0.88 (s each, 3H each, 7×Me); ^13^C-NMR (pyridine-*d*_5_): *δ* 174.8 (C-28), 145.4 (C-13), 121.8 (C-12), 104.9 (C-1'), 101.8(C-1''), 88.8 (C-3), 75.9 (C-2'), 74.1, 73.9 (C-3', C-4''), 72.6, 72.4 (C-2'', C-3''), 69.9 (C-5''), 68.7(C-4'), 64.8 (C-5'), 56.0 (C-5), 55.6 (C-piperazine), 48.1 (C-9), 47.6, 46.7 (C-17, C-19), 46.0, 45.7, 44.1 (C-piperazine), 42.2, 42.2 (C-14, C-18), 39.6, 39.5, 38.9 (C-4, C-8, C-1), 37.1 (C-10), 34.2 (C-21), 33.4 (C-29), 33.2, 33.2 (C-7, C-22), 30.6 (C-20), 30.2 (N*C*H_3_), 28.3, 28.1 (C-15,C-23), 26.5 (C-2), 26.2 (C-27), 24.2, 23.8, 22.8 (C-30, C-11, C-16), 18.6 (C-6, C-6''), 17.3, 17.0 (C-26, C-24), 15.7 (C-25); HRMS: calcd for C_4__6_H_76_N_2_O_10_ (M+Na): 839.5500; found: m/z 839.5497.

#### 3.6.10. 1-morpholinoole-28-one 3-O-α-L-rhamnopyranosyl-(1→2)-α-L-arabinopyranoside (***TS10***)

White powder, m.p. 201.7–204.1ºC; [α]^25^_D_ -10.1 (*c *0.10, CH_3_OH); ^1^H-NMR (pyridine-*d*_5_): *δ* 6.16 (s, 1H, H-1''), 5.40 (br s, 1H, H-12), 4.88 (d, 1H, *J* = 4.9 Hz, H-1'), 4.73 (m, 1H, H-2''), 4.64–4.55 (m, 3H, H-2', H-3'', H-5''), 4.32–4.26 (m, 4H, H-3', H-4', H-5'-1, H-4''), 3.84–3.68 (m, 9H, H-5'-2, H-morpholine), 3.38 (dd, 1H, *J* = 11.5, 4.0 Hz, H-3), 3.24 (br d, 1H, *J* = 7.9 Hz, H-18), 1.62 (d, 3H, *J* = 5.7 Hz, H-6''), 1.25, 1.18, 1.09, 0.94, 0.93, 0.89, 0.88 (s each, 3H each, 7×Me); ^13^C-NMR (pyridine-*d*_5_): *δ* 175.0 (C-28), 145.2 (C-13), 122.0 (C-12), 105.0 (C-1'), 101.8 (C-1''), 88.8 (C-3), 75.9 (C-2'), 74.1, 74.0 (C-3', C-4''), 72.6, 72.5 (C-2'', C-3''), 69.9 (C-5''), 68.8 (C-4'), 67.2 (C-morpholine), 64.9 (C-5'), 56.0 (C-5), 48.1 (C-9), 47.6 (C-morpholine), 46.6, 46.4 (C-17, C-19), 44.1 (C-morpholine), 42.2 (C-14, C-18), 39.6, 39.5 (C-4, C-8), 38.9 (C-1), 37.1 (C-10), 34.2 (C-21), 33.3 (C-29), 33.2 (C-7, C-22), 30.6 (C-20), 30.0 (C-morpholine), 28.2, 28.1 (C-15, C-23), 26.6 (C-2), 26.2 (C-27), 24.2 (C-30), 23.8, 22.7 (C-11, C-16), 18.6 (C-6, C-6''), 17.2, 17.0 (C-26, C-24), 15.7 (C-25); HRMS: calcd for C_4__5_H_73_NO_1__1_ (M+Na): 826.5184; found: m/z 826.5180.

#### 3.6.11. N-phenyloleanolic amide 3-O-α-L-rhamnopyranosyl-(1→2)-α-L-arabinopyranoside (***TS11***)

White powder, m.p. 156.7–158.3ºC; [α]^25^_D_ -11.9 (*c *0.15, CH_3_OH);^ 1^H-NMR (pyridine-*d*_5_): *δ* 7.76–7.29 (m, 5H, Ar-H), 6.13 (s, 1H, H-1''), 5.55 (br s, 1H, H-12), 4.88 (d, 1H, *J = *5.4 Hz, H-1'), 4.73 (m, 1H, H-2''), 4.60–4.52 (m, 3H, H-2', H-3'', H-5''), 4.32–4.28 (m, 4H, H-3', H-4', H-5'-1, H-4''), 3.80 (d, 1H, *J = *11.1 Hz, H-5'-2), 3.22 (br d, 1H, *J* = 10.8 Hz, H-3), 3.02 (br d, 1H, *J* = 11.6 Hz, H-18), 1.60 (m, 3H, H-6''), 1.26, 1.14, 1.02, 0.96, 0.94, 0.77, 0.75 (s each, 3H each, 7×Me); ^13^C-NMR (pyridine-*d*_5_): *δ* 175.8 (C-28), 143.2 (C-13), 138.4, 127.4, 127.3, 127.0, 123.8, 123.7 (6×Ar-C), 122.5 (C-12), 104.2 (C-1'), 101.1 (C-1''), 88.1 (C-3), 75.2 (C-2'), 73.4, 73.2 (C-3', C-4''), 72.0, 71.8 (C-2'', C-3''), 69.3 (C-5''), 68.0 (C-4'), 64.0 (C-5'), 55.1 (C-5), 47.5, 47.2, 46.1 (C-9, C-17, C-19), 41.9 (C-14), 41.5 (C-18), 39.1 (C-4), 38.8 (C-8), 38.2 (C-1), 36.3 (C-21), 32.7 (C-29), 32.4, 32.1 (C-7, C-22), 30.2 (C-20), 27.4 (C-15), 27.1 (C-23), 25.8 (C-2), 25.5 (C-27), 23.6 (C-30), 23.2, 23.0 (C-11, C-16), 17.9, 17.8 (C-6, C-6''), 16.4, 16.3 (C-24, C-26), 14.9 (C-25); HRMS: calcd for C_4__7_H_71_NO_1__0_ (M+Na): 832.5078; found: m/z 832.5072.

#### 3.6.12. N-(2-hydroxyphenyl)oleanolic amide 3-O-α-L-rhamnopyranosyl-(1→2)-α-L-arabinopyranoside (***TS12***)

White powder, m.p. 191.3–194.0ºC; [α]^25^_D_ -28.6 (*c *0.21, CH_3_OH);^ 1^H-NMR (pyridine-*d*_5_): *δ* 12.27 (br s, 1H, Ph-O*H*), 9.07 (m, 1H, Ar-H), 8.98 (m, 1H, Ar-H), 7.13 (m, 1H, Ar-H), 7.00 (m, 1H, Ar-H), 6.14 (s, 1H, H-1''), 5.67 (br s, 1H, H-12), 4.87 (d, 1H, *J* = 5.3 Hz, H-1'), 4.72 (m, 1H, H-2''), 4.63–4.52 (m, 3H, H-2', H-3'', H-5''), 4.32–4.25 (m, 4H, H-3', H-4', H-5'-1, H-4''), 3.80 (br d, 1H, *J* = 9.6 Hz, H-5'-2,), 3.20 (dd, 1H, *J* = 11.6, 4.1 Hz, H-3), 3.07 (br d, 1H, *J* = 13.2 Hz, H-18), 1.60 (d, 3H, *J* = 6.1 Hz, H-6''), 1.26, 1.13, 1.02, 0.91, 0.89, 0.82, 0.70 (s each, 3H each, 7×Me); ^13^C-NMR (pyridine-*d*_5_): *δ* 175.7 (C-28), 147.2 (OH-Ph-C), 143.3 (C-13), 128.2 (Ar-C), 123.6, 123.4 (Ar-C, C-12), 119.9 (Ar-C), 119.3 (Ar-C), 114.8 (Ar-C), 104.2 (C-1'), 101.1 (C-1''), 88.1 (C-3), 75.2 (C-2'), 73.4, 73.2 (C-3', C-4''), 71.9, 71.8 (C-2'', C-3''), 69.2 (C-5''), 68.0 (C-4'), 64.1 (C-5'), 55.1 (C-5), 47.4, 47.3, 46.3 (C-9, C-17, C-19), 42.0 (C-14), 41.6 (C-18), 39.1 (C-4), 38.8 (C-8), 38.3 (C-1), 36.2 (C-10), 33.8 (C-21), 32.8, 32.4, 32.2 (C-7, C-22, C-29), 30.2 (C-20), 27.4 (C-15), 27.2 (C-23), 25.9 (C-2), 25.4 (C-27), 23.8, 23.3, 23.0 (C-11, C-16, C-30), 17.9, 17.8 (C-6, C-6''), 16.3, 16.2 (C-24, C-26), 14.9 (C-25); HRMS: calcd for C_4__7_H_71_NO_1__1_ (M+Na): 848.5027; found: m/z 848.5019.

#### 3.6.13. N-benzyloleanolic amide 3-O-α-L-rhamnopyranosyl-(1→2)-α-L-arabinopyranoside (***TS13***)

White powder, m.p. 168.3–171.1ºC; [α]^25^_D_ -9.5 (*c *0.19, CH_3_OH); ^1^H-NMR (pyridine-*d*_5_): *δ* 8.02–8.00 (m, 1H, N*H*), 7.45 (m, 2H, Ar-H), 7.31 (m, 2H, Ar-H), 7.22 (m, 1H, Ar-H), 6.17 (s, 1H, H-1''), 5.37 (br s, 1H, H-12), 4.86 (d, 1H, *J = *5.4 Hz, H-1'), 4.77 (m, 2H, H-2'', NHC*H*_2_Ph), 4.63–4.55 (m, 4H, H-2', H-3'', H-5'', NHC*H*_2_Ph), 4.31–4.25 (m, 4H, H-3', H-4', H-5'-1, H-4''), 3.80 (m, 1H, H-5'-2), 3.22 (dd, 1H, *J* = 11.5, 4.1 Hz, H-3), 3.14 (br d, 1H, *J* = 13.9 Hz, H-18), 1.61 (d, 3H, *J = *6.6 Hz, H-6''), 1.23, 1.19, 1.07, 0.88, 0.88, 0.87, 0.85 (s each, 3H each, 7×Me); ^13^C-NMR (pyridine-*d*_5_): *δ* 177.5 (C-28), 144.8 (C-13), 140.9, 128.8, 128.8, 128.1, 128.1, 127.1 (Ar-C), 122.8 (C-12), 104.9 (C-1'), 101.8 (C-1''), 88.7 (C-3), 75.9 (C-2'), 74.1, 74.0 (C-3', C-4''), 72.6, 72.4 (C-2'', C-3''), 69.9 (C-5''), 68.8 (C-4'), 64.8 (C-5'), 55.8 (C-5), 47.9 (C-9), 46.9, 46.5 (C-17, C-19), 43.5 (*C*H_2_Ph), 42.1, 41.9 (C-14, C-18), 39.7 (C-4), 39.5 (C-8), 38.8 (C-1), 37.0 (C-10), 34.4 (C-21), 33.8 (C-7), 33.2 (C-29), 33.0 (C-22), 30.9 (C-20), 28.0, 27.9 (C-15, C-23), 26.5 (C-2), 26.1 (C-27), 23.7 (C-30), 23.7 (C-11), 23.7 (C-16), 18.6, 18.5 (C-6, C-6''), 17.4 (C-26), 17.0 (C-24), 15.6 (C-25); HRMS: calcd for C_4__8_H_73_NO_1__0_ (M+Na): 846.5234; found: m/z 846.5229.

## 4. Conclusions

In summary, 13 novel amide derivatives of β-hederin were synthesized and evaluated *in vitro* for tumor cytotoxicity. The results from the antitumor screening showed that compounds **TS8** and **TS9 **possessed potent antitumor activity against HeLa and MCF-7 cell lines. The preliminary structure-activity relationships indicated that the conversion of the C-28 carboxylic acid group of β-hederin into an amide derivative often resulted in a loss of broad spectrum antitumor activity, but also resulted in an increase in the antitumor selectivity. To provide more clarity about the structure-activity relationship, further studies on additional systematic structural variations are underway. 
